# Optimization of Cellulose Derivative-, PVA-, and PVP-Based Films with *Reynoutria japonica* Extract to Improve Periodontal Disease Treatment

**DOI:** 10.3390/ma17246205

**Published:** 2024-12-19

**Authors:** Arleta Dołowacka-Jóźwiak, Izabela Nawrot-Hadzik, Adam Matkowski, Piotr Nowakowski, Ruth Dudek-Wicher, Dorota Markowska, Robert Adamski, Dorota Krzyżanowska-Gołąb, Bożena Karolewicz

**Affiliations:** 1 Department of Drug Form Technology, Wroclaw Medical University, Borowska 211 A, 50-556 Wroclaw, Polandbozena.karolewicz@umw.edu.pl (B.K.); 2 Department of Pharmaceutical Biology and Biotechnology, Division of Pharmaceutical Biology and Botany, Wroclaw Medical University, 50-556 Wroclaw, Poland; izabela.nawrot-hadzik@umw.edu.pl (I.N.-H.); bbsekret@umw.edu.pl (A.M.); 3 Department of Pharmaceutical Microbiology and Parasitology, Faculty of Pharmacy, Wroclaw Medical University, 50-367 Wroclaw, Poland; ruth.dudek-wicher@umw.edu.pl; 4 Faculty of Process and Environmental Engineering, Lodz University of Technology, 90-924, Lodz, Poland; dorota.siuta@p.lodz.pl (D.M.); robert.adamski@p.lodz.pl (R.A.); 5 Department of Chemistry and Immunochemistry, Wroclaw Medical University, M. Sklodowskiej-Curie 48/50, 50-369 Wroclaw, Poland; dorota.krzyzanowska-golab@umw.edu.pl

**Keywords:** *Reynoutria japonica* extract, cellulose derivatives, resveratrol, polyvinyl alcohol, polyvinylpyrrolidone, carriers in dental local healing

## Abstract

The aim of this study was to develop and optimize polymeric films based on cellulose derivatives—hydroxypropylmethylcellulose (HPMC), methylcellulose (MC), and sodium carboxymethylcellulose (NaCMC)—as well as pullulan, polyvinyl alcohol (PVA), polyvinylpyrrolidone (PVP), and glycerol (GLY) as plasticizer incorporating *Reynoutria japonica* extract for potential use in periodontal and gum disease treatment. Over 80 formulations were fabricated using the solvent-casting method, 6 of which were selected for further investigation based on their mechanical properties, mucoadhesion, and disintegration profiles, including three placebo films (OP1 (PVA/PVP/MC400CP/NaCMC/GLY), OP2 (PVA/PVP/MCA15C/NaCMC/GLY), and OP3 (PVA/PVP/HPMC/NaCMC/GLY)) and three films containing *R. japonica* extract (OW1, OW2, and OW3). The films demonstrated uniform structural characteristics, with the formulations containing PVA with a high hydrolysis degree (98–99%) and methylcellulose derivatives showing prolonged dissolution times due to physical cross-linking, while the inclusion of NaCMC reduced dissolution time without compromising mucoadhesiveness. The study also described the release kinetics of resveratrol and piceid from the OW2 films using three semi-empirical models: the Korsmeyer–Peppas model, a first-order kinetic model, and a multidimensional approach. The multidimensional model demonstrated a strong fit, with a correlation coefficient (R^2^) of 0.909 for resveratrol, compared to 0.894 and 0.908 for the Korsmeyer–Peppas and first-order models, respectively. For piceid, the multidimensional model showed a correlation coefficient (R^2^) of 0.958, outperforming the Korsmeyer–Peppas (0.823) and first-order models (0.932). The active compounds released in sustained-release tests, including resveratrol and piceid, suggest that these films could provide an extended therapeutic effect.

## 1. Introduction

In dentistry, one of the most significant challenges is the effective treatment of oral diseases, which are among the most prevalent noncommunicable diseases globally, affecting approximately 3.5 billion people each year [1]. Although the quality of care for all oral disorders shows an increasing trend on a global scale, oral health conditions still rank among the top 10 of all diseases globally and have a negative impact on the global economy. The annual expenditure was estimated at USD 387 billion in direct costs and another USD 323 billion in indirect costs. In terms of indirect costs, severe periodontal disease generated USD 82 billion in expenses [1,2,3]. The complexity of oral diseases, compounded by individual patient variability, the multifunctionality of the oral cavity, and its inherent mobility, complicates the development of drugs with optimal physicochemical properties that can meet therapeutic needs and enhance treatment efficacy [3,4].

The primary objectives of oral disease therapy are to protect and regenerate damaged tissues and mitigate inflammation, which can lead to the formation of pathological pockets within the oral cavity. Crucial considerations in drug design include ensuring safety, ease of administration, convenient application, and targeted release of the active substance at the site of action, thereby reducing dosing frequency [4,5].

The escalating rates of antimicrobial resistance (AMR) pose a significant global health concern, primarily driven by the overutilization of antifungal agents and antibiotics. This trend underscores the imperative for intensified research into novel substances exhibiting antimicrobial, antioxidant, and anti-inflammatory properties. The study of medicinal substances of natural origin, mainly processed medicinal plants, is of great interest to the scientific community. Moreover, medicinal plants are an essential source of bioactive compounds and antioxidant substances for the human body [6]. Currently, local herbal therapy is successfully used, and according to the World Health Organization (WHO), over 80% of the global population prefers herbal remedies, highlighting these natural substances’ significant role in healthcare today [7,8]. Rhizomes of *Reynoutria japonica* Houtt. (under the pharmacopoeial name *Polygoni cuspidati rhizoma et radix*, *hu zhang* in pinyin Chinese), used as a source of active substances, are plant medicinal raw materials whose use originated in traditional Chinese medicine. This herbal medicine was included in the European Pharmacopoeia in 2017. *R. japonica* (common name: Japanese Knotweed) belongs to the *Polygonaceae* family, and its natural habitat includes regions in East Asia [9,10,11]. In Europe, this plant is considered an invasive species, posing a threat to native plant species due to its ability to produce substances that inhibit the growth of other plants [11,12]. The extract obtained from the rhizome of *R. japonica* is rich in chemical substances from various groups, including stilbene derivatives like resveratrol and piceid—compounds known for their significant biological activities [13,14]. The high resveratrol content contributes to the anti-inflammatory and antipyretic effects of the extract. Additionally, resveratrol is known for its antioxidant properties, making it a valuable aid in combating various inflammatory conditions and infections [11,14]. Hence, this species may be a promising therapy in preventing and healing lesions in the oral cavity and treating periodontitis. Resveratrol is absorbed from food in the intestine through diffusion and carrier-mediated transport [15]. In enterocytes, resveratrol undergoes biotransformation, leading to the formation of well-soluble, easily excretable, and biologically active derivatives [16,17,18]. Interestingly, a specific metabolism of resveratrol was observed in cancer cells. They overexpress the cytochrome P450 enzyme CYP1B1, which catalyzes aromatic hydroxylation reactions. These cells hydroxylate resveratrol at positions 4 and/or 3’, producing three metabolites, one of which is piceatannol (3,5,3’,4’-tetrahydroxystilbene), a known anticancer and antileukemic agent, which is being considered as a potential anticancer drug [19]. A major resveratrol glucosylated derivative—piceid—has antimicrobial effects against oral pathogens, which are important in preventing dental diseases. It has been shown to inhibit the biofilm formation of Fusobacterium nucleatum, a bacterium associated with periodontal diseases [20]. Furthermore, piceid exhibits antimicrobial effects against both planktonic and biofilm forms of *Porphyromonas gingivalis*, another key pathogen in periodontal disease [20].

Our research team conducted extensive research to select the best extract from *R. japonica rhizomes* to develop a polymer film for potential use in the treatment of periodontal and gingival diseases [21,22]. For the development of the film, we selected a 25% ethanolic extract which effectively stimulated human gingival fibroblasts to proliferate, migrate, and increase the synthesis of collagen III. This activity, according to the phytochemical analysis, may be related to the high contents of resveratrol and piceid and the relevant composition of procyanidins.

Modern carriers of active pharmaceutical substances, including fibers, mucoadhesive strips and films, gels, liposome systems, microparticles, nanoparticles, and nanofibers, play a vital role in periodontology. Film carriers can be applied to the surface of the oral mucosa in the area of the cheeks and gums but can also be applied directly to the gum pockets.

A considerable advantage of this type of pharmaceutical form is its suitability for cutting out smaller fragments of a specific size and shape tailored to the individual needs of the patient. Their flexibility allows them to be easily placed in the desired location, unlike other conventional pharmaceutical forms, such as mucoadhesive tablets. Adhesion to the oral mucosa makes polymer films preferred over oral solutions or oral gels, which are usually easily removed by saliva and therefore have a shorter residence time on the mucosa. The mucoadhesive properties do not allow the film to be easily swallowed or inhaled, which minimizes the risk of choking.

Recently, films based on a mixture of natural and synthetic polymers enriched with plant extracts have gained significant popularity in biomedical applications. Cellulose and its derivatives are widely used in pharmaceutical and periodontal formulations due to properties such as biocompatibility, biodegradability, nontoxicity, film-forming ability, the possibility of chemical modification, chemical stability, good moisture retention capacity, and flexibility in processing [23,24,25]. Their mucoadhesive properties allow for prolonged contact time with the specific tissues, thus enhancing bioavailability and preventing too rapid degradation for drug delivery applications [26]. At the nanoscale, cellulose displays distinct adhesion properties that play a critical role in its interactions with microbial surfaces. These nanoscale properties enable cellulose to effectively bind to bacterial cells, thereby modulating bacterial adherence and influencing biofilm formation dynamics [27,28].

However, the limitations of cellulose- and cellulose derivative-based carriers, such as insufficient mechanical strength and barrier properties, pose significant challenges. To mitigate these shortcomings, blending cellulose and its derivatives with other water-soluble polymers, such as polyvinyl alcohol (PVA), is a promising approach to improve polymer-based carrier thermomechanical and morphological properties, including enhanced tensile and modulus properties. PVA is a highly valuable polymer in dental applications due to its nontoxicity, high crystallinity, water solubility, biodegradability, flexibility, hydrophilicity, mucoadhesive properties, controlled tensile strength, and excellent film-forming ability [29]. It does not exhibit mutagenic or clastogenic effects, ensuring that it does not cause genetic mutations or chromosomal damage. Additionally, PVA does not accumulate in the body, further supporting its safety profile [30]. In our study, to ensure appropriate mucoadhesive properties [31,32,33,34] and to extend the release time of substances incorporated into the carrier, additional polyvinylpyrrolidone (PVP) was included in the composition of the polymeric film formulation. PVP aids in reducing the periodontal pocket depth and improving connective tissue attachment in deep periodontal pockets, thereby reducing the number of pathogenic microorganisms in the periodontium [35,36].

Considering that polymer films prepared with *R. japonica* extract have not yet been investigated in dental applications, the optimal contents and proportions of these composite films to achieve better mechanical and bioactive properties remain unclear. This study aimed to develop the composition, preparation technology, and assessment of the physico-chemical properties of polymer films based on cellulose derivatives. The tested substances included hydroxypropylmethylcellulose (HPMC), methylcellulose (MC), sodium carboxymethylcellulose (NaCMC), pullulan, PVA, and PVP containing 25% ethanolic extract from rhizomes of *R. japonica* as promising compositions for use on the oral mucosa in the treatment of periodontal and gum diseases [37].

The researchers hypothesize that it will be possible to develop carriers with the required release profile through advanced cross-linking processing. This approach will serve as a disintegration-rate modeling technology to predict the release profile and facilitate the selection of therapies based on clinical needs. Null Hypothesis (H₀): The release of resveratrol and piceid from the polymeric films does not follow the multidimensional model. This hypothesis was tested by fitting the drug release data to the multidimensional equation.

## 2. Materials and Methods

### 2.1. Materials

Polyvinyl alcohol (PVA—Mwt: 85,000–124,000, degree of hydrolysis: 98–99%; Mwt: 31,000–50,000, degree of hydrolysis: 85–89%, CAS: 9002-89-5), polyvinylpyrrolidone (PVP, K90—Mwt: 40,000, CAS: 9003-39-8), methylcellulose (MC 400 cP—viscosity of 2% aqueous solution at 20 °C: 300–560 cP, CAS: 9004-67-5; A15C, Methocel^®^ (Pharmaceutical Institute, Warsaw, Poland), viscosity of 2% aqueous solution at 20 °C: 1200–1800 mPa.s, CAS: 9004-67-5), hydroxypropylmethylcellulose (HPMC—viscosity of 2% aqueous solution at 20 °C: 40–60 cP, CAS: 9004-65-3), mucin from pig stomachs (Type III—bound to sialic acid: 0.5–1.5%, CAS: 84082-64-4), and pullulan (CAS: 9057-02-7) were acquired from Sigma-Aldrich (Sigma-Aldrich, MO, USA). Carboxymethyl cellulose sodium salt (NaCMC—viscosity of 2% aqueous solution at 25 °C: 300–600 mPa.s, CAS: 9004-32-4) was purchased from VWR Chemicals (Modlniczka, Poland). Glycerol (GLY—Glycerolum 85%, batch no.: 081056), obtained from Fagron (Modlniczka, Poland), was used for the preparation of the films. Ethyl alcohol (96% *v*/*v*, CAS: 64-17-5) was purchased from Honeywell (Modlniczka, Poland). Resveratrol, a standard substance (≥98%, serial no.: N811.3), was acquired from Roth (Modlniczka, Poland). All chemicals used in the study were of analytical grade and used as received without further purification unless otherwise mentioned.

The rhizomes of *Reynoutria japonica* with a diameter of 15–30 mm used in this study were harvested in September in urban habitats near the city of Wroclaw, Poland, and were deposited in the herbarium of the Botanical Garden of Medicinal Plants, Department of Pharmaceutical Biology and Botany of the Medical University of Wrocław. Klemens Jakubowski, M.Sc. in Botany from the Botanical Garden of Medicinal Plants herbarium, identified the species.

### 2.2. Methods

#### 2.2.1. Preparation of Reynoutria Japonica Extracts

The plant extract from the rhizome of *Reynoutria japonica* was prepared according to the procedure described in [33,34]. Fifty grams of air-dried and powdered rhizomes of *R. japonica* was used for the extraction process. The powdered rhizomes were extracted with 500 mL of 25% ethanol. Extraction was performed using an ultrasonic bath for 2 h. After extraction, the solvent was evaporated under reduced pressure to obtain the dried extracts.

#### 2.2.2. Film Preparation

**Figure 1:**
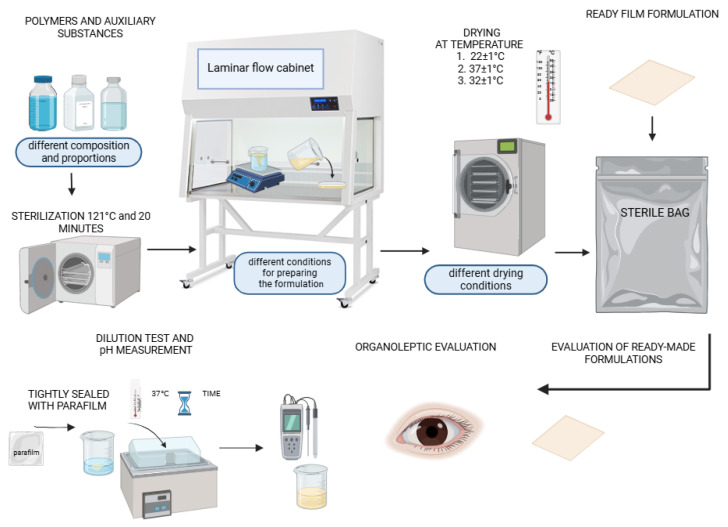
Schematic of preparation procedure for cellulose derivative-based and PVA/PVP/pullulan-based composite *placebo* film formulations.

Film formulations were developed utilizing the solvent-casting technique within a controlled laminar flow cabinet. This method is recognized for its ability to produce uniform films with desirable mechanical and physical properties, making it suitable for various applications, including drug dosage forms. A total of 82 series (from A to I, provided in the Supplementary Materials) of polymer film formulations were fabricated, each varying in the composition of key ingredients, such as polyvinyl alcohol with degrees of hydrolysis of 98–99 and 85–89% (2.5%, 5%, and 10%), polyvinylpyrrolidone (5% and 10%), hydroxypropyl methylcellulose (2.5% and 5%), methylcellulose (MC 400 cP—1%, 3%, and 5%; MC A15C—1%, 3%, and 5%), pullulan (2.5% and 5%), , sodium carboxymethyl cellulose (4%), and glycerol (0.15 g, 0.25 g, 0.4 g, 3 g, 4 g, and 5 g). The variations in the formulations were designed to explore the effects of different polymer combinations and preparation conditions on the resulting film properties. Figure 1 shows a schematic procedure for obtaining a composite film preparation.

**Figure 2:**
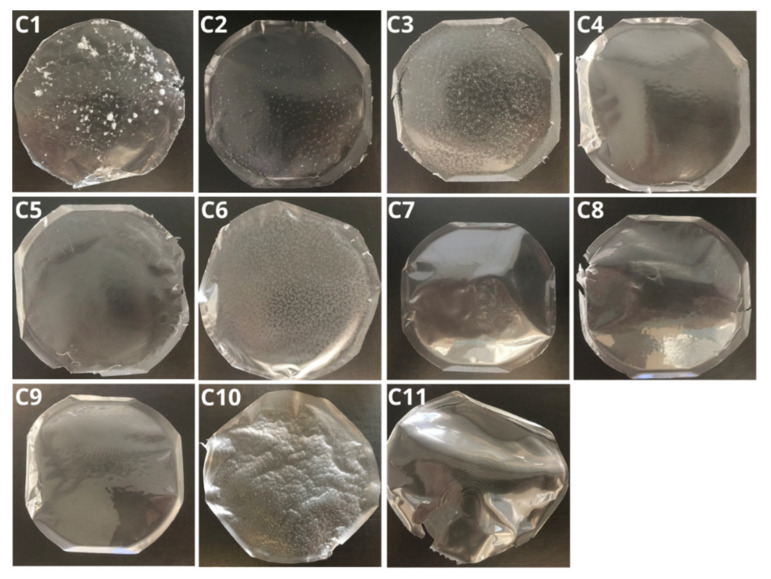
Series C1–C11 of the produced polymeric films with 0.15 g GLY: C1 (50/50 10% PVA, 3% MC A15C), C2 (50/50 10% PVA, 1.5% MC A15C), C3 (50/50 10% PVA, 3% MC A15C), C4 (50/50 10% PVA, 1% MC 400), C5 (50/50 5% PVA, 3% MC 400), C6 (50/50 5% PVA, 1.5% MC 400), C7 (50/50 10% PVA, 2.5% pullulan), C8 (50/50 10% PVA, 2,5% HPMC), C9 (50/50 10% PVA, 2.5% PVP), C10 (50/50 5% PVA, 1.5% MC A15C), and C11 (50/50 5% PVA, 5% pullulan). In all formulations, PVA had a degree of hydrolysis of 98–99%.

An example series of the fabricated polymeric films is shown in Figure 2. All 82 produced films were evaluated for elasticity, brittleness, color, transparency, gloss, and the presence or absence of polymer component agglomerates or hardened air bubbles within the structure. The use of PVA with a hydrolysis degree of 98–99%, along with an increased content of methylcellulose derivatives (MC 400CP and MCA 15C), contributed to prolonged dissolution times due to physical cross-linking processes. In contrast, the inclusion of NaCMC effectively shortened the dissolution time while maintaining the films’ mucoadhesive properties. Developed polymer films with air bubbles require longer degassing and cooling of the mixture at 4–8 °C to improve the homogeneity of the structure. Increasing the glycerol content to 3–5% improves the elasticity of the film, while reducing this value makes it possible to obtain stiffer materials adapted to specific applications. The different compositions of films affect their dissolution time, allowing the design of supports with adapted properties and applications in the treatment of mucous membranes, gums, the tongue, and periodontal pockets in dentistry. Table 1 presents example results for the evaluation of films C1–C11.

Out of the initial 82 formulations, 3 optimized variants were selected based on their qualitative characteristics, such as elasticity, brittleness, color, transparency, gloss, presence or absence of polymer component agglomerates, and absence of hardened air bubbles in the structure. All optimized films demonstrated morphological and structural uniformity, indicating the precise selection of ingredients and the effectiveness of the mixing and blending techniques for the polymer mixtures. The uniform morphologies also confirmed that the casting and drying conditions were appropriately controlled.

**Figure 3:**
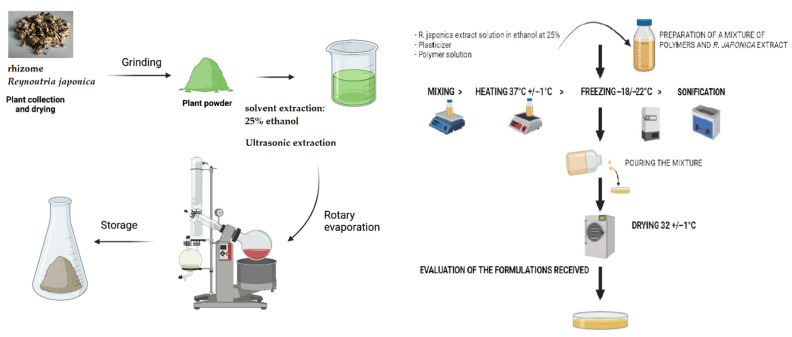
The process of fabricating films with *R. japonica* extract.

The detailed compositions of these selected formulations are outlined in Table 2. For this purpose, 3 mL of an aqueous–ethanol solution (with a water/ethanol ratio of 25:75 m/m) containing 100 mg of dry *R. japonica* extract previously micronized in an agate mortar was introduced into the prepared 50 g OP1-OP3 formulations. Before being incorporated into the formulations, the prepared aqueous–ethanolic extract solution was filtered through a syringe filter with a pore diameter of 0.22 µm (PTFE; Millex Samplicity^®^ Filters, Merck KGaA, Germany). After the addition of the extract solution, the formulations were mixed using a magnetic stirrer with a simultaneous heating function (Labinco LD846; Labinco BV, The Netherlands). Finally, the specified volume of glycerol was added to the mixture during stirring. The mixture was sonicated (Advantage-Lab GmbH, type: AL-04-12; UK) to remove air bubbles until a clear solution was obtained. The thus-prepared liquid formulations were poured in amounts of 45 g into polystyrene dishes with an area of 92 cm^2^ and dried in a laboratory drier (Pol-Eko-Aparatura Sp. J. Type: SLN 115 simple; Poland) at a temperature of 32 ± 1 °C for 48 h. After drying and removal from the dishes, the films were stored in sterile zip-up bags (Chemivet, Poland) at room temperature for further testing. A diagram illustrating the stage of preparing the formulations containing *R. japonica* extract is presented in Figure 3. The dried films were divided into 25 × 25 mm inserts, and samples were stored in the dark in a sealed desiccator until further analyses.

#### 2.2.3. Physical Property Characterization of Films

The fabricated films were evaluated for thickness and mass uniformity. The thickness of the obtained films was measured at different points using a screw micrometer gauge (Hard Head, USA). For mass measurements, samples (3 per film) were cut into squares (25 mm × 25 mm) and weighed on an analytical scale (Pioneer PA2/02CM/1; Ohaus Corp., Parsippany, NJ, USA). For mass uniformity, cut samples were weighed individually using an electronic balance (Pioneer; Ohaus Corp., Parsippany, NJ, USA), and their weights were recorded as the means of three measurements.

##### Mechanical Property Testing

Tests of the mechanical properties of the film formulations were carried out based on the method described by Vecchi et al. [38]. For the study, film fragments measuring 2.5 cm × 2.5 cm were prepared, which were repeatedly folded at 180° at the same point in the formulation until they cracked and broke, or until 300 folds were obtained without film breakage. Three fragments cut from different locations of the polymer film were measured for each formulation.

##### Mucoadhesion Test

The study was conducted on 2.5 cm × 2.5 cm sections of the polymeric films using a TA.XT Plus Texture Analyzer equipped with a movable arm and an A/Muc mucoadhesion test probe (Stable Micro System, Godalming, UK). For the measurement, a 13 mm mucin disc was prepared by compressing 250 mg of powdered mucin using a hydraulic press (Specac, 15t; Godalming, UK) with a 13 mm die (Specac 13 mm DIE PT. No 3000; Godalming, UK) attached to the probe arm using double-sided adhesive tape under a pressure of 10 tons for 30 s. Prior to the mucoadhesion test, the mucin disc was moistened with a 5% aqueous mucin solution. The probe arm with the attached mucin disc was then lowered at a speed of 1 mm/s into a beaker containing artificial saliva, and the film sample was mounted in the A/Muc fixture. The beaker, along with the submerged A/Muc fixture, was placed on a magnetic stirrer with heating capability, ensuring continuous mixing, and the solution was maintained at a temperature of 37 ± 1 °C throughout the measurement. Upon contact between the mucin disc and the film surface, a force of 0.1 N was applied for 30 s, after which the arm was programmed to lift at a speed of 1 mm/s, measuring the force required to detach the mucin disc from the film surface [39]. The test was repeated for three polymer film samples taken from different areas of the dry formulation.

##### Disintegrating Time

Disintegration time was reported as the time required for film samples to fully dissolve. For measurements, film samples were cut into squares (25 mm × 25 mm) and incubated at 37 °C in 10 mL of water. Sealed vessels were horizontally shaken (60 cycles per minute) in a heated shaking batch (Memmert S1422; Memmert GmbH, Schwabach, Germany).

##### pH Film Studies

pH measurements of extracts obtained after blurring the film fragments were carried out by the potentiometric method using a pH meter (CPC–511; Elmetron, Zabrze, Poland) with a combined electrode (ERH-12-6 no.: 2071) at a temperature of 22 ± 1 °C with an accuracy of 0.01 pH units.

##### Contact Angle Measurement

A 50 μL water drop was carefully dispensed onto the surface of the film. This small volume was chosen to minimize the impact of gravity on the shape of the water droplet, ensuring that the contact angle measurement reflected the surface properties of the film rather than external forces. The interaction between the water drop and the film surface was recorded with a precision of up to 0.01° using the Ossila Contact Angle System (Sheffield, UK) equipped with a high-resolution camera and software capable of capturing and analyzing the shape of the water droplet in real time. After the water drop was applied, the height and width of the droplet on the film surface were measured. These dimensions were crucial for determining the shape of the droplet, which was directly related to the contact angle. The contact angle value was automatically calculated by the system’s software.

#### 2.2.4. Chemical Structure

Analysis of the pure polymers (PVA, PVP, MC, HPMC, and NaCMC), *R. japonica* extract, resveratrol, and selected films was performed using an FTIR/ATR (Fourier Transform Infrared Spectroscopy/attenuated total reflection) spectrometer (Thermo Nicolet iS50; Waltham, MA, USA) [40]. IR spectra were measured in the 500–4000 cm^−1^ range at a resolution of 4 cm^−1^, a scan speed of 0.2 cm/s, and 32 scans per sample.

#### 2.2.5. Study of the Release Profile of Resveratrol and Piceid from the Selected Polymer Films Containing R. Japonica Extract

The release profile analysis of *R. japonica* extract components was conducted in a thermostatically controlled water bath (Memmert model: WB22; Büchenbach, Germany) at a temperature of 37 ± 1 °C with horizontal shaking capability. Film fragments measuring 2.5 cm × 2.5 cm were accurately weighed and placed in beakers tightly sealed with Parafilm containing 10 mL of purified water at 37 ± 1 °C. The prepared beakers were transferred to the water bath with horizontal shaking at a speed of 60 cycles per minute. Samples for active substance determination were taken in volumes of 1.0 mL at 1, 3, 5, 7, 12, and 24 h, and each time the withdrawn volume was replenished with 1.0 mL of purified water at 37 ± 1 °C using an automatic pipette. The collected samples were subsequently dried in a solution concentration apparatus (VLM GmbH model: V.659.061.820; Büchenbach, Germany) under a stream of liquid nitrogen. To the dried samples, 1.0 mL of a methanol–water solution (80:20, *v*/*v*) was added, and the samples were shaken for 1 h. After this period, the obtained solutions were filtered through a syringe filter with a pore diameter of 0.22 µm (PTFE Millex Samplicity^®^ Filters; Büchenbach, Germany).

The prepared solutions were then subjected to qualitative and quantitative analysis using high-performance liquid chromatography–mass spectrometry (LC-MS). The method described by [33,34] was employed to determine the contents of resveratrol and piceid released from the film fragments containing *R. japonica* extract.

Qualitative and quantitative analysis of the release of *R. japonica* extract components from the developed polymer films was conducted using the Ultimate 3000RS series system (Thermo Dionex, Sunnyvale, CA, USA), which was equipped with a low-pressure quaternary gradient pump, a vacuum degasser, an autosampler, a column compartment, and DAD (Diode Array Detection). The system was further integrated with a high-resolution quadrupole time-of-flight mass spectrometer (Bruker qTOF Compact; Bruker Daltonik, Billerica, MA, USA) equipped with electrospray ionization (ESI). The analytical column used was a Kinetex C18 with dimensions of 2.6 μm (150 mm × 2.1 mm), maintained at a temperature of 30 °C. The mobile phase consisted of two components: A (H_2_O: HCOOH, 100:0.1, *v*/*v*) and B (acetonitrile: HCOOH, 100:0.1, *v*/*v*). The gradient program was set as follows: 0–22 min with 15–22% B, 22–33 min with 22–95% B, followed by column equilibration with 15% B for 2 min between injections. The flow rate was maintained at 0.3 mL/min, and the analysis of samples was repeated four times to ensure accuracy. UV–Vis spectra were recorded in the range of 200–450 nm, with chromatograms acquired at 298 nm. The mass spectrometer was equipped with ESI, operating under specific conditions: splitless mode, nebulizer pressure at 30 psi, dry gas flow at 8 L/min, dry temperature at 250 °C, and capillary voltage set at 2.2 kV for negative-ion mode and 4.5 kV for positive-ion mode. Mass spectra were recorded over a scan range of m/z 50–2200, with collision energy automatically adjusted between 20 and 40 eV based on the m/z of the fragmented ions.

#### 2.2.6. Statistical Analysis

All assays were conducted in at least triplicate, and obtained results are presented as the means of the replicates ± SDs. The two-way ANOVA test (GraphPad Prism v. 9, San Diego, CA, USA) was used to assess significant differences between the obtained values. The statistical software OriginLab 2020, Northampton, MA, USA was utilized to fit the kinetic equations, while nonlinear multivariate optimization was employed to estimate the coefficients. The objective function was defined as the squared difference between the experimental data and the results predicted by the kinetic model.

## 3. Results and Discussion

### 3.1. Visual Assessment

**Figure 4:**
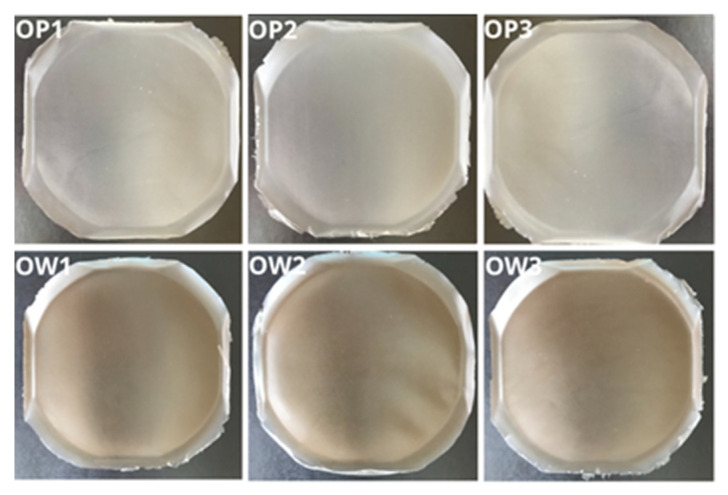
Photographic documentation of the optimized films: placebo series (OP1, OP2, and OP3) and those containing the plant extract from *R. japonica* (OW1, OW2, and OW3).

All developed polymer film formulations containing the extract from *R. japonica*, namely, OW1, OW2, and OW3, demonstrated consistent morphological and structural properties (Figure 4). No evidence of polymer aggregates, delaminations, or trapped air bubbles was observed within any of the formulations, indicating a high degree of uniformity. The films were notably flexible, nonsticky, easy to remove from molds, and exhibited a smooth surface. The inclusion of *R. japonica* extract in the OW-series formulations imparted a slight brownish tint to the films. Apart from this color variation, no significant organoleptic differences were observed between the OW films and the placebo series (OP1, OP2, and OP3).

### 3.2. Thickness and Mass Measurements of Polymer Films

The evaluation of film thickness and mass is a critical parameter for determining the structural integrity and uniformity of polymeric film formulations. In this study, the thickness and mass of polymer films were measured to assess the reproducibility and uniformity of the manufacturing process across different formulations. Table 3 presents the average thickness and mass values for fragments of each formulation, along with the calculated standard deviations (SDs).

The results of the measurements indicated that the OW1 formulation, containing the plant extract from *R. japonica*, and the placebo formulation OP1, both with the same polymer-based composition of film, exhibited the greatest average thickness. In contrast, the OW2 formulation and its placebo counterpart, OP2, demonstrated the lowest average thickness. These differences are a result of compositional differences. OW1 and OW3 were prepared using an aqueous solution of MC 400 cP at a 5% concentration (polymer ratio of 5:95), while OW2 and OP2 were formulated using an aqueous solution of MC A15C at a 3% concentration (polymer ratio of 3:97). The thickness of OW3 was similar to OW1, attributed to the use of a 5% aqueous HPMC solution (polymer ratio of 5:95). No significant differences in the average thickness were observed between the films containing *R. japonica* extract and their respective placebo formulations. Additionally, the calculated standard deviations for thickness across all formulations were lower than the precision threshold of the measurement device, indicating a high degree of uniformity in film thickness. Regarding mass measurements, the OW2 and OP2 formulations exhibited the lowest average mass, while OW1 and OP1 exhibited the highest. These results were consistent with the thickness measurements, as formulations with the lowest average thickness also had the lowest mass, and those with the greatest thickness had the highest mass. This correlation was due to the different concentrations of methylcellulose derivatives used in the formulations. The standard deviations for mass measurements across all formulations ranged from 2.00 to 2.45, indicating a comparable level of uniformity and reproducibility in the preparation method across all tested films.

### 3.3. Mechanical Bending Resistance Test of Polymer Films

The mechanical bending resistance of the polymer films was evaluated using a standardized method. All prepared formulations, including placebo films and those containing the plant extract from *R. japonica*, exhibited high resistance to bending. In all three repetitions for each formulation, the films withstood at least 300 bends without tearing or breaking. This demonstrates the optimal mechanical properties and high durability of the produced polymer films. The results indicate that the inclusion of the plant extract did not compromise the mechanical integrity of the films, suggesting that the formulations maintain their structural performance even with the addition of active components.

### 3.4. Mucoadhesion Strength Test

Mucoadhesion is a crucial parameter in evaluating polymeric carriers as dosage forms for application on the oral mucosa. Table 4 presents the average mucoadhesion strength values, along with the standard deviations for the three placebo film formulations (OP1, OP2, and OP3) and three formulations containing *R. japonica* extract (OW1, OW2, and OW3).

The results indicate that the highest average mucoadhesion strength values were obtained for the OP2 and OW2 formulations, while the lowest values were recorded for the OW3 and OP3 formulations. The OW2 and OP2 formulations contained MCA15C with a viscosity range of 1200–1800 mPa·s, whereas the OW3 and OP3 formulations contained HPMC with a viscosity range of 20–40 mPa·s. The higher viscosity of MCA15C contributed to the increased mucoadhesion strength of the films containing this polymer. Furthermore, all measurements were characterized by low standard deviation values, indicating the reproducibility of the applied film production method.

### 3.5. Wettability Test

Wettability is a critical parameter in evaluating the performance of polymer films, especially in applications such as oral mucosal delivery systems. The degree of wettability affects how a film behaves upon contact with biological fluids, such as saliva or gingival crevicular fluid, influencing both the rate of hydration and the subsequent dissolution of the active ingredients embedded within the film matrix. Films with lower contact angles are likely to absorb more fluid, potentially enhancing the release and diffusion of the active compounds at the site of application. The contact angle measurements, presented in Table 5, indicate that all the polymer film formulations exhibit contact angles below 90°, signifying partial wettability. This suggests that the films possess a hydrophilic surface, as evidenced by the ability of water to spread to some degree across the surface. Notably, no statistically significant differences in contact angle values were observed between the films containing *R. japonica* extract (OW series) and their placebo counterparts (OP series). The lowest contact angle values were recorded for the placebo formulation OP1 and its corresponding extract-containing film OW1, while the highest contact angles were associated with formulations OP3 and OW3. This trend in wettability can be directly attributed to the intrinsic hydrophilicity of the polymers used in the formulations. Specifically, the lower contact angles suggest that these films, particularly OP1 and OW1, may be more inclined to interact with and absorb moisture from their environment compared to the other formulations.

### 3.6. Effect of Syringe Filter Membrane on the Content of R. Japonica Extract Components

The results of the study shown in Table 6 highlighted substantial variations in the measured contents of the key components of *R. japonica* extract, namely, resveratrol and piceid, depending on the type of filter membrane employed in the filtration process. Notably, the use of a nylon filter resulted in a marked depletion of filtrate in both resveratrol and piceid. The resveratrol content was reduced to approximately 2.11% of the declared value, while piceid levels in the filtrate were below the detection limit of the analytical method employed. In contrast, the PVDF filter with a pore size of 0.45 µm led to an increase in resveratrol content, surpassing the declared content, with values exceeding 100% of the expected dose. This phenomenon reflects the retention or less efficient filtration of resveratrol through this membrane, possibly due to its molecular weight or solubility characteristics. Additionally, the PVDF filter with a 0.22 µm pore size exhibited a significant reduction in resveratrol content, down to approximately 20.87% of the declared dose, while the piceid content was moderately decreased to around 76.64%. The detailed HPLC-RI chromatograms of resveratrol and piceid released from the *R. japonica* extract depending on the type of filter membrane employed in the filtration process are provided in the Supplementary Materials.

These results suggest that membrane pore size and material composition play a pivotal role in the selective retention or loss of extract components during the filtration process. The observed differences underscore the necessity of carefully selecting the appropriate filter membrane when preparing plant extracts for analysis or formulation development, as filtration can substantially influence the integrity and composition of the extract. The use of inappropriate membranes may lead to the loss of key bioactive components, thereby affecting the reproducibility and reliability of subsequent analyses and applications of the extract.

### 3.7. Determination of Film Dissolution Time and pH Value of Extracts After Dissolution

Table 7 presents the average values obtained from three measurements of dissolution time for film fragments and the pH values of extracts after dissolution of placebo and film formulations containing *R. japonica* extract.

The results of the study confirmed the extended dissolution time of the optimized polymeric films. Formulations OW1 and OW2 achieved complete dissolution within 24 h, whereas films from formulation OW3 were dissolved within 20 h. No significant differences were observed between the plant-extract-containing films from *R. japonica* and their *placebo* counterparts. The pH values of the extracts obtained after the dissolution of the film fragments ranged from 7.15 for formulation OW1 to 7.32 for formulation OW3. The obtained appropriate pH values of the extracts, close to a neutral value, in the planned application are beneficial for ensuring carrier tolerance and reducing the risk of irritation at the application site.

### 3.8. Fourier Transform Infrared Spectroscopy (FT-IR)

**Figure 5:**
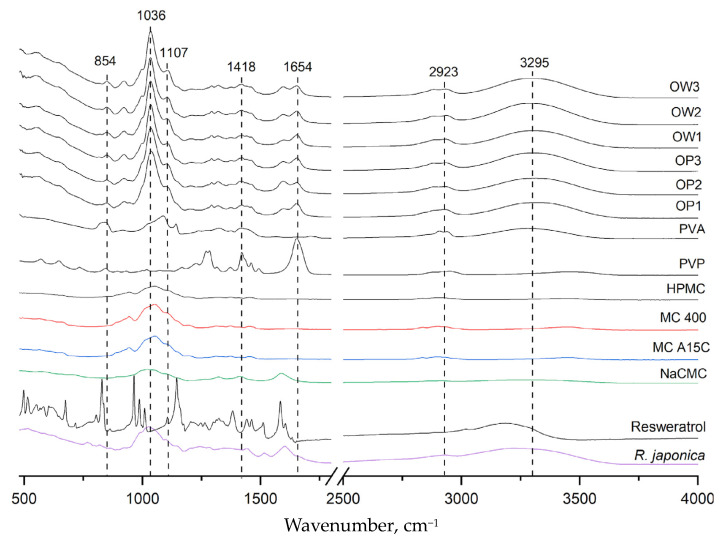
FT-IR spectra for all excipients, plant extracts, resveratrol, and prepared polymer film formulations.

Figure 5 presents the infrared absorption spectra for all polymer components: polyvinyl alcohol (PVA), polyvinylpyrrolidone (PVP), methylcellulose (MC), hydroxypropyl methylcellulose (HPMC), sodium carboxymethylcellulose (NaCMC), powdered *Reynoutria japonica* extract, pure resveratrol standard, placebo films (OP1, OP2, and OP3), and three film formulations containing *R. japonica* extract (OW1, OW2, and OW3). The PVA spectrum exhibited a characteristic band in the range of 3000–3500 cm⁻^1^, centered around 3295 cm⁻^1^, corresponding to the stretching vibrations of hydroxyl (OH) groups involved in intra- and intermolecular hydrogen bonding. The PVP spectrum showed a peak at 1654 cm⁻^1^, which is associated with the stretching vibrations of the carbonyl (C=O) bonds in the pyrrolidone ring. Additionally, a broad absorption band between 1050 and 1430 cm⁻^1^ was observed, corresponding to the stretching vibrations of C-O bonds and the bending of -CH₂ and -CH groups. This broad band is prominent in the spectra of MC, NaCMC, and HPMC, indicating their structural similarity in this region. In the FT-IR spectra of the placebo formulations (OP1, OP2, and OP3) and the films containing *R. japonica* extract (OW1, OW2, and OW3), no new absorption bands were observed beyond those characteristic of the formulation components. The 3295 cm⁻^1^ band confirmed the presence of PVA, while the 1654 cm⁻^1^ band corresponded to PVP. The broad band in the range of 1036–1418 cm⁻^1^ was attributed to methylcellulose or its derivatives. The FT-IR results indicate a lack of interactions between the extract components and the polymers in the film.

### 3.9. In Vitro Release Profile of Resveratrol and Piceid from PVA/PVP/MCA15C/NaCMC Films with the R. Japonica Extract

**Figure 6:**
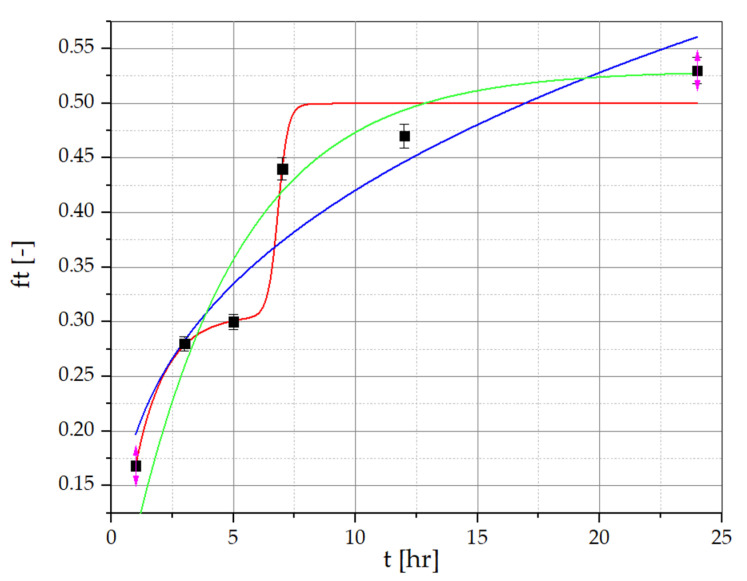
Resveratrol release data for PVA/PVP/MCA15C/NaCMC film layers with *R. japonica* extract. ▪ Experimental points (statistically significant with *p* ≤ 0.05) approximated with the Korsmeyer–Peppas model (green line), the multidimensional model (red line), and the first-order kinetic model (blue line).

**Figure 7:**
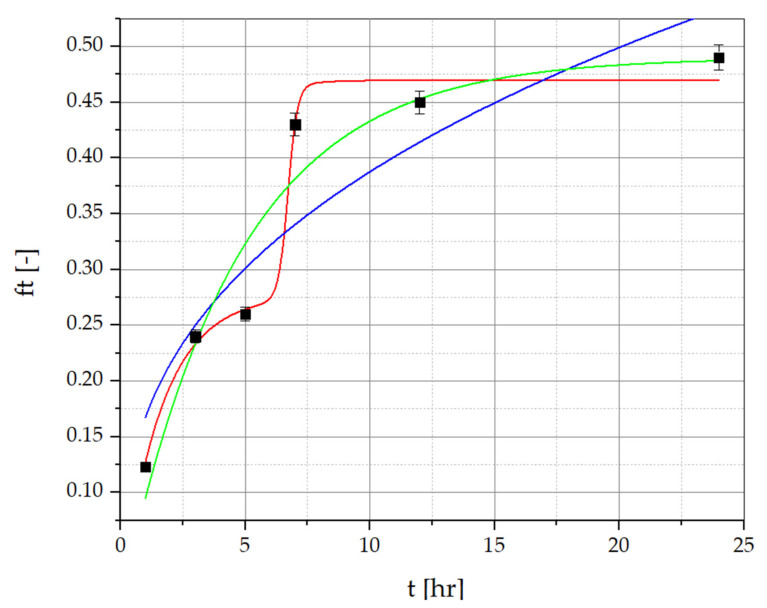
Piceid release data for PVA/PVP/MCA15C/NaCMC film layers with *R. japonica* extract. ▪ Experimental points (statistically significant with *p* ≤ 0.05) approximated with the Korsmeyer–Peppas model (green line), the multidimensional model (red line), and the first-order kinetic model (blue line).

To determine the amount of resveratrol and piceid released from the developed PVA/PVP/MCA15C/NaCMC/GLY formulations containing *R. japonica* extract, the method described by Nawrot-Hadzik et al. [33,34] was employed. The detailed HPLC-RI chromatograms of resveratrol and piceid determined for the films and the *R. japonica* extract are provided in the Supplementary Materials. The results of the release study for the optimized OW2 film series are presented in Figure 6 and Figure 7, showing the percentage of resveratrol and piceid released at 1, 3, 5, 7, 12, and 24 test hours. The concentrations of these compounds were determined using calibration curves based on analytical-grade standards of pure resveratrol and piceid. The release profile of resveratrol from the OW2 polymeric film exhibited an initial rapid release, commonly referred to as the ‘burst effect’, with approximately 26.78% of the total dose released within the first 3 h. This was followed by a deceleration in the release rate until the fifth hour, after which a renewed increase was observed, reaching 44.33% by the seventh hour. This phase is characterized by a diffusion-controlled release mechanism, where the concentration gradient between the polymer matrix and the surrounding medium drives the migration of resveratrol molecules from the interior to the exterior of the matrix. The final stage of resveratrol release involves matrix erosion, during which the polymer gradually degrades or dissolves, resulting in the continuous release of the remaining resveratrol. This leads to a prolonged, gradual release phase lasting up to 24 h, culminating in the release of 51.26% of the total resveratrol dose. The piceid released from the OW2 polymeric film followed a similar pattern, with an initial rapid release of 25.63% within the first 3 h, followed by a noticeable deceleration until the fifth hour. After this period, the release rate increased again, reaching 43.29% by the seventh hour. In the subsequent phase, a significant slowdown in the release process was observed, with 46.63% of the total dose released by the 24th hour.

Three types of semi-empirical kinetic models, including the Korsmeyer–Peppas model (Equation (1)), a first-order model (Equation (2)), and a multidimensional model (Equation (3)), were used to explain the release mechanisms of resveratrol and piceid.

(1) $$ft=a\cdot t^{n}$$

(2) $$ft={ft}_{max}\cdot \left({1-e}^{-k_{1}\cdot t}\right)$$

(3) $$ft=k_{5}\cdot \left({1-e}^{-k_{1}\cdot t}\right)+k_{4}\cdot \frac{e^{k_{2}\cdot t-k_{3}}}{\left({1+e}^{k_{2}\cdot t-k_{3}}\right)}$$

where

- *ft*—the fraction of resveratrol or piceid released at time *t*;
- *ft_max_*—the maximum fraction of substance released during the process;
- *a*—the kinetic constant of the Korsmeyer–Peppas equation that is dependent on the structural and geometric characteristics of the drug–polymer system;
- *n*—the exponent defining the mechanism of the drug release;
- *k*_1_–*k*_5_—kinetic constants.

The Korsmeyer–Peppas equation is frequently applied to describe drug release from polymeric systems, particularly when the release mechanism is not fully known or involves multiple processes. This model is most accurate when the percentage of drug released is below 60%, providing insight into both diffusion-controlled and anomalous transport mechanisms [41,42,43,44,45]. Meanwhile, the first-order rate equation is useful for systems where the release rate is directly proportional to the concentration of the drug remaining in the formulation. This model effectively describes cases in which the release is concentration-dependent, making it suitable for water-soluble drugs or systems exhibiting first-order release [44,45].

Resveratrol and piceid release data for PVA/PVP/MCA 15C/NaCMC film layers, approximated with the Korsmeyer–Peppas, first-order kinetic, and multidimensional models, are presented in Figure 6 and Figure 7, and correlation coefficients (R^2^) of the regression equation are collected in Table 8 and Table 9. The regression coefficient (R^2^) values obtained from the first-order kinetic model are greater than those obtained from the Korsmeyer–Peppas model in the case of resveratrol and piceid released from OP2 film layers. The best fitting of the curves is presented by the multidimensional kinetic model.

### 3.10. Analysis of the Content of R. Japonica Extract in a Single Polymer Carrier

The content analysis of resveratrol and piceid in the *R. japonica* extract was performed on three distinct fragments of the OW2 carrier (a, b, and c) based on the procedure presented in Section 2.2.5, each fragment measuring 2.5 cm × 2.5 cm and sourced from different areas of the film. This specific formulation was chosen due to its superior performance in the release study, where it was demonstrated that it released the highest concentrations of bioactive compounds over time. Table 10 provides a detailed account of the masses of the film fragments analyzed, the theoretical contents of resveratrol and piceid (calculated using established calibration curves for each substance), and the percentages of released compounds, along with the mean values and standard deviations.

The analysis revealed that the average resveratrol content in the dry OW2 film fragments was 36.31% of the theoretical dose. The standard deviation, ±6.16%, indicates significant variability in the measurements, pointing to potential procedural limitations. This suggests that improvements in the quantification method are necessary, particularly with respect to sample filtration post dissolution of the carrier.

Similarly, the measured piceid content was approximately 30.44% of the theoretical dose, with a standard deviation of ±6.55%. This considerable variability highlights the need for further refinement of the experimental protocols, including potential adjustments to improve accuracy and consistency in the analysis of both resveratrol and piceid contents.

The null hypothesis (H₀) stating that the release of resveratrol and piceid from the polymeric films does not follow the multidimensional model was rejected. This conclusion is supported by the obtained results, which demonstrate favorable mechanical properties, optimal mucoadhesion, and a sustained release profile for the active compounds, indicating the potential applicability of these films for the treatment of periodontal diseases.

### 3.11. Limitations of the Study

Although our studies offer valuable insights, it is important to acknowledge several limitations that warrant consideration. Firstly, although over 80 formulations were initially developed, only six (OP1–OP2 and OW1–OW3) were selected for detailed analysis. This selection may not have adequately captured the full spectrum of performance differences across all variants, such that potentially promising alternatives may have been overlooked. Moreover, the emphasis on these selected formulations may have led to the neglect of other critical factors, such as the stability of the films and their behavior under long-term storage conditions. Another limitation lies in the in vitro nature of the experiments. While these controlled conditions allow for precise analysis, they do not fully replicate the complexities of the human oral environment, where variables such as saliva composition, enzymatic activity, and oral microflora significantly influence the performance of polymer films. The absence of clinical studies further exacerbates this issue, restricting the ability to validate the therapeutic efficacy of the polymer films in the treatment of periodontal disease and hindering their application in practical healthcare settings. Lastly, the study does not address potential adverse effects or allergic reactions associated with the use of polymer films. These factors are critical for assessing the overall safety profile of the proposed treatment and represent a significant gap in the current research.

## 4. Conclusions

In this study, over 80 polymeric films incorporating cellulose derivatives, PVA, PVP, and pullulan were developed (series A to I) using the solvent-casting method. Ultimately, six formulations—the placebos OP1 (PVA/PVP/MC 400C P/NaCMC/GLY), OP2 (PVA/PVP/MCA 15C/NaCMC/GLY), and OP3 (PVA/PVP/HPMC/NaCMC/GLY) and OW1, OW2, and OW3 containing *R. japonica* extract—were selected. The created films exhibited uniform morphological and structural properties, optimal mechanical and mucoadhesive characteristics, and extended disintegration times, demonstrating suitability as potential active substance carriers for periodontal disease therapy.

The method for analyzing the components of *R. japonica* extract in the liquid obtained after the dissolution of the polymer films was also found to be important. The choice of filtration medium was crucial for obtaining accurate results regarding the content of resveratrol and piceid, two active ingredients in the extract. In a 24 h pharmaceutical availability test, the optimized formulations of films containing *R. japonica* extract achieved prolonged release of the active ingredients. This suggests that these film formulations may be effective in delivering these active ingredients over an extended period, potentially improving their therapeutic efficacy. Moreover, during the film release studies, it was observed that not only the main compound, resveratrol, was released from the *R. japonica* extract, but also piceid. This unexpected result emphasizes the need for further studies to better understand the release mechanism and potential interactions. Additionally, efforts should be directed toward optimizing the concentration of *R. japonica* extract and other cellulose derivatives to assess their impact on film properties and bioactivity, as well as exploring the specific biological mechanisms through which the active compounds in *R. japonica* contribute to periodontal healing. Further studies are necessary to assess the pharmaceutical usefulness of the developed formulations and their in vivo efficacy.

## Figures and Tables

**Table 1:** Results of the selection criteria for polymer films of the C1-C11 series.

Formulation	C1	C2	C3	C4	C5	C6	C7	C8	C9	C10	C11
Polymer agglomerates	+	+	+	-	+	+	-	-	-	+	-
Air bubbles	+	+	+	-	-	+	-	-	-	-	-
Elasticity	-	-	-	-	-	-	-	-	+	+	-
Transparency	+	+	-	+	-	+	+	+	+	-	+
Gloss	+	+	-	+	-	-	+	+	+	-	+
Ease of removal from the mold	+	+	+	+	+	+	+	+	+	+	+
Disintegration time [min]	35.00	12.00	24.00	14.00	45.00	19.00	3.00	23.00	5.00	22.00	2.00
pH after disintegration	6.70 ± 0.02	6.67 ± 0.02	6.71 ± 0.02	6.72 ± 0.02	6.61 ± 0.02	6.58 ± 0.02	6.65 ± 0.02	6.79 ± 0.02	6.53 ± 0.02	6.72 ± 0.02	6.59 ± 0.02

“+” indicates the presence or a positive result for a given parameter; “-” indicates the absence or a negative result for a given parameter.

**Table 2:** The selected optimized composition of fabricated films with *R. japonica* extract (OW) and placebo (OP).

Components of the Film Formulations	OP1	OP2	OP3	OW1	OW2	OW3
PVA (degree of hydrolysis of 98–99%) [g]	11.00	11.00	11.00	11.00	11.00	11.00
PVP [g]	6.50	6.50	6.50	6.50	6.50	6.50
MC 400 cP [g]	17.50	-	-	17.50	-	-
MC A15C [g]	-	17.50	-	-	17.50	-
HPMC [g]	-	-	17.50	-	-	17.50
NaCMC [g]	15.00	15.00	15.00	15.00	15.00	15.00
GLY [g]	1.50	1.50	1.50	1.50	1.50	1.50
Water–ethanol (25:75 m/m) solution with 100 mg *of R. japonica* extract [mL]	-	-	-	3.00	3.00	3.00
Water–ethanol (25:75 m/m) [mL]	3	3	3	-	-	-

**Table 3:** Results of thickness and mass measurements of optimized polymer films.

Formulation Designation	Average Thickness [nm] ± SD	Mean Mass [mg] ± SD
OP1	256.01 ± 5.47	183.45 ± 2.00
OW1	258.32 ± 4.47	184.23 ± 2.45
OP2	202.11 ± 4.47	161.67 ± 2.18
OW2	208.12 ± 4.47	163.00 ± 2.37
OP3	238.35 ± 4.47	180.13 ± 2.24
OW3	244.48 ± 5.47	181.69 ± 2.15

**Table 4:** Mucoadhesion measurements of optimized polymer films containing *R. japonica* extract.

Formulation Designation	Mean Mucoadhesion [g] ± SD
OP1	199.47 ± 0.60
OW1	199.13 ± 0.50
OP2	270.07 ± 0.75
OW2	270.10 ± 0.36
OP3	190.30 ± 0.75
OW3	188.90 ± 0.38

**Table 5:** Contact angle measurements of optimized polymer films.

Formulation Designation	Average Contact Angle [◦] ± SD
OP1	50.78 ± 0.35
OW1	50.68 ± 0.32
OP2	59.03 ± 0.26
OW2	58.63 ± 0.31
OP3	60.05 ± 0.59
OW3	61.84 ± 0.31

**Table 6:** Summary of filters used in the study of membrane effects on the content of *R. japonica* extract components after filtration.

Filter Type	PTFE 0.22 [µm]	PVDF 0.22 [µm]	MC 0.22 [µm]	NYLON 0.22 [µm]	PVDF 0.45 [µm]
Declared piceid content [µg]	26.11	26.11	26.11	26.11	26.11
Measured piceid content [µg]	24.33	20.01	24.22	0.55	23.96
Piceid content [%]	93.18	76.64	92.76	2.11	91.76
Declared resveratrol content [µg]	3.45	3.45	3.45	3.45	3.45
Measured resveratrol content [µg]	3.32	0.72	3.28	-	3.62
Resveratrol content [%]	96.23	20.87	95.07	-	104.93

**Table 7:** Results of the dissolution time and pH analysis after dissolution of optimized polymer films.

Formulation Designation	Average Dissolution Time [h]	Average pH ± SD
OP1	24	7.17 ± 0.02
OW1	24	7.15 ± 0.02
OP2	24	7.19 ± 0.01
OW2	24	7.20 ± 0.02
OP3	20	7.28 ± 0.02
OW3	20	7.32 ± 0.01

**Table 8:** Kinetic parameters for resveratrol release from OP2 film layers.

Kinetic Parameters	Korsmeyer–Peppas Model	First-Order Model	Multidimensional Model
a	0.197 ± 0.02	-	-
${ft}_{max}$	-	0.53	-
*n*	0.329 ± 0.06 (Fickian diffusion)	-	-
*k*_1_	-	0.225 ± 0.02	0.799 ± 0.57
*k*_2_	-	--	4.86 ± 4.20
*k*_3_	-		33.23 ± 2.45
*k*_4_	-	-	0.194 ± 0.09
*k*_5_	-	-	0.306 ± 0.08
R^2^	0.894	0.908	0.909

**Table 9:** Kinetic parameters for piceid release from OP2 film layers.

Kinetic Parameters	Korsmeyer–Peppas Model	First-Order Model	Multidimensional Model
a	0.168 ± 0.03	-	-
${ft}_{max}$	-	0.49	-
*n*	0.364 ± 0.08 (Fickian diffusion)	-	-
*k*_1_	-	0.215 ± 0.02	0.613 ± 0.43
*k*_2_	-	--	5.217 ± 6.70
*k*_3_	-		35.06 ± 12.9
*k*_4_	-	-	0.192 ± 0.09
*k*_5_	-	-	0.277 ± 0.08
R^2^	0.823	0.932	0.958

**Table 10:** Identification and contents of individual components of the *R. japonica* extract in polymer film fragments from the different OW2 formulations (a, b, and c).

Formulation Designation	OW2a	OW2b	OW2c	Average Value ± SD
Fragment mass [mg]	163.36	167.40	165.18	165.31 ± 2.02
Theoretical piceid content [µg]	128.20	131.38	129.63	129.74 ± 1.59
Measured piceid content [µg]	38.17	31.84	48.35	39.46 ± 8.33
Piceid content [%]	29.77	24.24	37.30	30.44 ± 6.55
Theoretical resveratrol content [µg]	17.12	17.54	17.31	17.33 ± 0.21
Measured resveratrol content [µg]	5.82	5.55	7.49	6.29 ± 1.05
Resveratrol content [%]	33.97	31.66	43.28	36.31 ± 6.16

## Data Availability

The data presented in this study are available on request from the corresponding author.

## Supplementary Materials

The results of the cellulose-, cellulose derivative-based, and PVA/PVP/pullulan-based composite film formulations for series A to I can be downloaded at https://www.mdpi.com/article/10.3390/ma17246205/s1.
