# Supplementary material for: Optimization of Cellulose Derivative-, PVA-, and PVP-Based Films with Reynoutria japonica Extract to Improve Periodontal Disease Treatment
Source: Materials (Basel). 2024 Dec 19;17(24):6205. doi: 10.3390/ma17246205 (PMC11677500; doi:10.3390/ma17246205)
Supplement: Supplementary file 1 [file materials-17-06205-s001.zip › materials-3310603-supplementary.pdf]

Preliminary analysis of polymer films made from a single type of polymer

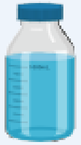

Preliminary selection of casting process parameters i
Drying and evaluation of physico-chemical properties
of the films.

Formulation designation	Composition of the solution
A1	10% PVA 72 000 cP
A2	5% PVA 98-99% hydrolizy
A3	5% PVP
A4	1 % MC 400 cP
A5	3% MC 400 cP
A6	1% MC 1500 cP
A7	5% HPMC
A8	10% HPMC
A9	2,5% Pullulan
A10	5% Pullulan
A11	4% NaCMC

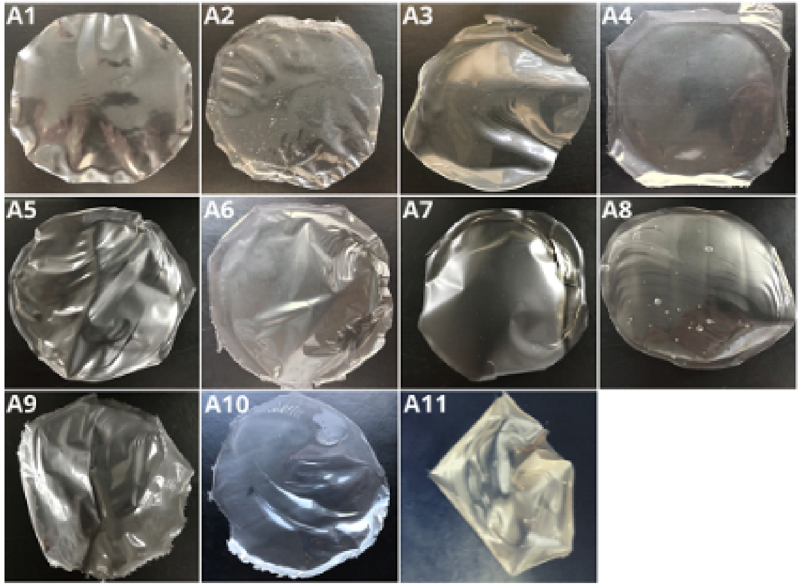

Formulation designation	A1	A2	A3	A4	A5	A6	A7	A8	A9	A10	A11
Polymer agglomerates	-	-	-	-	-	-	-	-	-	-	-
Bubbles of air	-	+	-	+	-	-	+	+	-	-	+
Flexibility	-	-	-	-	-	-	-	-	-	-	-
Clear/transparent	+	+	+	+	+	+	+	+	+	+	-
Gloss	+	+	+	+	+	+	+	+	+	+	-
Easy to remove from the mold	+	+	+	+	+	+	+	+	+	+	+
Disintegration time [min]	30	120	6	12	120	10	5	35	2	6	20
pH	6,41	6,95	6,48	7,20	7,19	7,21	7,14	7,09	7,05	7,11	7,41

To summarise the main experimental steps involved in the preparation and initial evaluation of polymer films from two-component blends of polymer solutions, taking into account detailed parameters of physico-chemical analysis and evaluation of film-forming properties.

1. Purpose:
 a. To check the behaviour of solutions during mixing and pouring.
 b. To analyse the morphological and structural homogeneity of the films after drying.

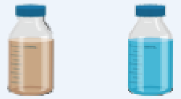

2. Film Preparation Process:
 a. Preparation of individual polymer solutions as outlined in Table in a laminar flow hood
 b. Mixing of polymer solutions in a 50:50 weight ratio
 c. Casting 30 g of the mixture into polystyrene dishes with a surface area of 92 cm²
 d. Drying at room temperature for 72 hours

Formulation designation	Composition of the solution	
B1	10% PVA	2,5%Pullulan
B2	5% PVA	5% Pullulan
B3	2,5%Pullulan	5% PVP
B4	5% Pullulan	5% HPMC
B5	5% PVP	2,5% HPMC
B6	5% HPMC	3% MC1500
B7	2,5% HPMC	1,5% MC1500
B8	3% MC1500	1% MC 1500
B9	1,5% MC1500	3% MC400
B10	1% MC 1500	3% MC400
B11	3% MC400	1,5% MC400

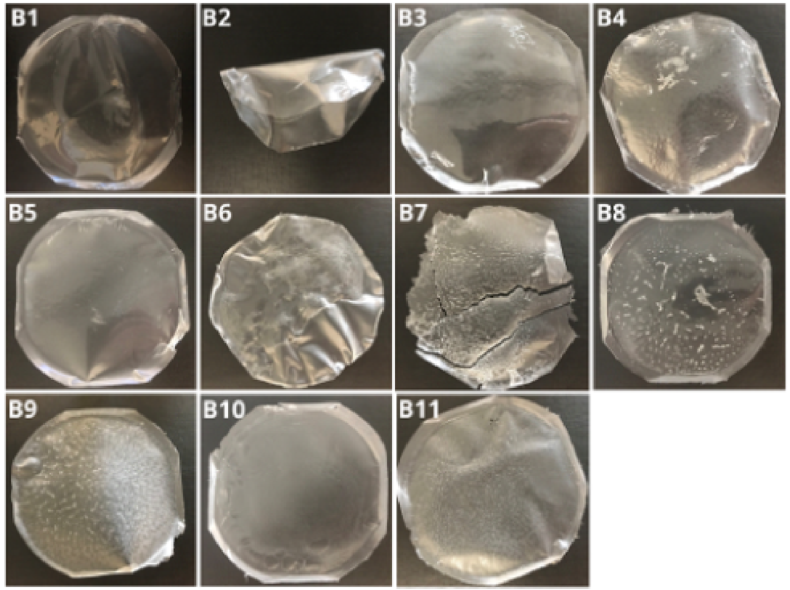

Formulation designation	B1	B2	B3	B4	B5	B6	B7	B8	B9	B10	B11
Polymer agglomerates	+	-	-	+	+	+	+	+	+	+	+
Bubbles of air	-	-	-	-	+	-	-	+	+	-	+
Flexibility	-	-	-	-	-	-	-	-	-	-	-
Clear/transparent	+	+	+	+	+	+	-	+	-	-	-
Gloss	+	+	+	+	+	+	+	+	-	-	-
Easy to remove from the mold	+	+	+	+	+	+	-	+	+	+	+
Disintegration time [min]	3	10	8	24	20	28	18	10	24	34	15
pH	6,67	6,69	6,59	6,73	6,78	6,83	6,80	6,75	6,76	6,75	6,71

This scheme highlights the key stages and parameters of the experiment for the preparation and preliminary evaluation of polymer films using two polymers and the addition of a plasticiser, taking into account detailed parameters of physico-chemical analysis and evaluation of film forming properties.

1. Purpose:

a. Preparation of films from two-component polymer mixtures with the addition of glycerol.
b. Preliminary evaluation of film formation and physico-chemical properties of the films.

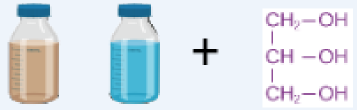

2. Film Preparation Process:

a. Preparation of polymer solutions according to table 10 in a laminar chamber.
b. Mix the polymer solutions in a 50:50 mass ratio.
c. Add 0.15g glycerol solution to the polymer mixture.
d. Pour 30 g of the mixture into polystyrene dishes with an 92 cm².
d. Drying at room temperature for 72 hours.

Formulation designation	Composition of the solution	
C1	10% PVA	3% MC1500
C2	10% PVA	1,5% MC1500
C3	10% PVA	3% MC400
C4	10% PVA	1% MC400
C5	5% PVA	3% MC400
C6	5% PVA	1,5% MC400
C7	10% PVA	2,5%Pullulan
C8	10% PVA	2,5% HPMC
C9	10% PVA	2,5% PVP
C10	5% PVA	5% Pullulan
C11	5% PVA	1,5% MC400

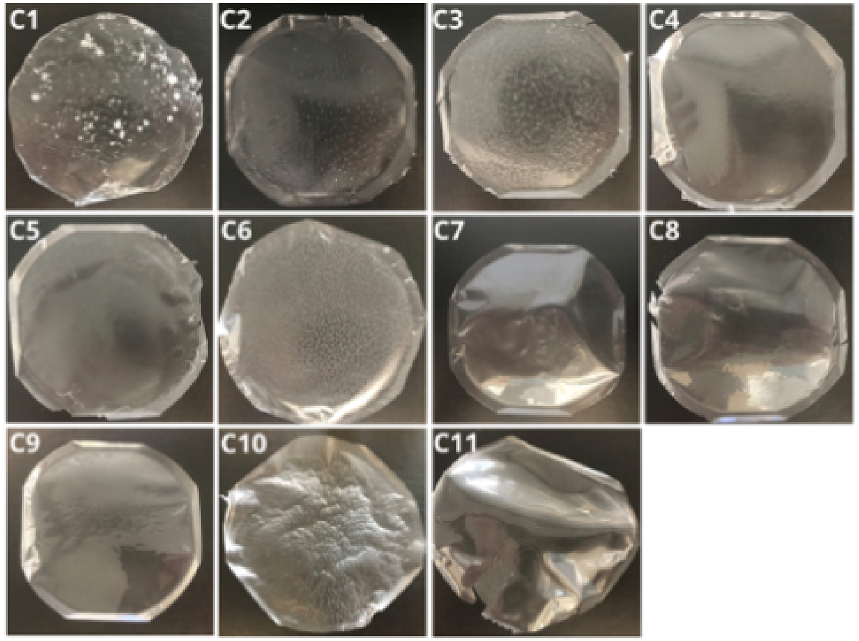

Formulation designation	C1	C2	C3	C4	C5	C6	C7	C8	C9	C10	C11
Polymer agglomerates	+	+	+	-	+	+	-	-	-	+	-
Bubbles of air	+	+	+	-	-	+	-	-	-	-	-
Flexibility	-	-	-	-	-	+	-	-	+	+	-
Clear/transparent	+	+	-	+	-	+	+	+	+	-	+
Gloss	+	+	-	+	-	-	+	+	+	-	+
Easy to remove from the mold	+	+	+	+	+	+	+	+	+	+	+
Disintegration time [min]	35	12	24	14	45	19	3	23	5	22	2
pH	6,71	6,67	6,71	6,72	6,61	6,58	6,65	6,79	6,53	6,72	6,59

This diagram clearly describes the process of making films from a mixture of polymers and the addition of a plasticiser, taking into account different casting conditions and the results of a preliminary assessment of their physical and chemical properties.

1. Purpose:

a. Preparation of films D1 - D10 from mixtures of two polymer solutions with the addition of 0.25 g of glycerol solution.
b. Preliminary evaluation of the properties of the films.

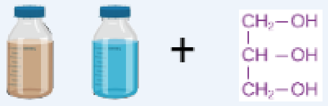

2. Film Preparation Process:

a. Prepare polymer solutions according to the table in a laminar chamber.
b. Mix the polymer solutions in the appropriate weight ratios.
c. Pour 10, 20 or 30 g of the mixture into different dishes:
- Formulations D1, D4, D10 for 92 cm² polystyrene dishes,
- Formulations D2, D3, D5, D8, D9 for 38,5 cm² glass Petri dishes,
- Formulations D6 and D7 for smaller 28,5 cm² polystyrene dishes.
d. Allow to dry for 72 hours at room temperature..

Formulation designation	Composition of the solution	
D1	10% PVA	2,5% PVP
D2	5% PVA	2,5% PVP
D3	2,5% PVP]	5% PVP
D4	5% PVP	1% MC400
D5	1% MC400	1% HPMC
D6	1% HPMC	2,5% HPMC
D7	2,5% HPMC	5% HPMC
D8	5% HPMC	2,5% HPMC
D9	2,5% Pullulan	2,5% Pullulan
D10	5% PVA	5% Pullulan

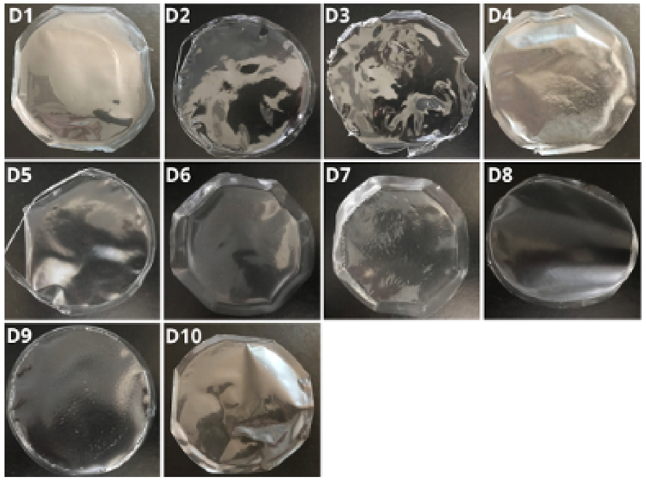

Formulation designation	D1	D2	D3	D4	D5	D6	D7	D8	D9	D10
Polymer agglomerates	-	-	-	+	-	-	+	-	+	-
Bubbles of air	-	-	-	-	-	+	+	-	+	-
Flexibility	-	-	-	+	+	-	-	+	-	-
Clear/transparent	+	+	+	-	-	+	+	+	-	+
Gloss	+	+	+	-	-	+	+	+	-	+
Easy to remove from the mold	30	10	10	30	30	10	10	20	20	30
Disintegration time [min]	5	5	5	23	21	7	21	22	35	2
pH	6,57	6,53	6,48	6,56	6,63	6,73	6,71	6,64	6,69	6,64

This diagram shows the process of manufacturing films using a mixture of polymers and the addition of a plasticiser, taking into account all the stages of production and the results of the initial assessment of the physico-chemical properties of the films.

1. Purpose:

a. Preparation of films E1 - E15 from mixtures of solutions of two or three polymer components with the addition of 4,0 g of glycerol solution.
b. Evaluation of the physico-chemical properties of the films, including the effect of the addition of a water-ethanol solution and the use of two cross-linking stages.

2. Fim Process:

a. Prepare polymer solutions according to the table in a laminar chamber.
b. Add 3 ml of water-ethanol solution (water:ethanol 25:75 m/m) to each formulation.
c. Mix the polymer solutions in the appropriate weight ratios.
d. Cross-linking by freezing and thawing the mixture twice at -18°C to -22°C with mixing between each step.
e. Pour 60 g of the mixture into polystyrene moulds of 92 cm² area.
f. Drying in a laminar air flow chamber for 72 hours.

Formulation designation	Composition of the solution		
E1	2,5% PVA	5% HPMC	
E2	2,5% PVA	5% HPMC	
E3	2,5% PVA	5% HPMC	
E4	2,5% PVA	5% HPMC	5% PVP
E5	2,5% PVA	5% HPMC	5% PVP
E6	2,5% PVA	5% MC400	
E7	2,5% PVA	5% MC400	
E8	2,5% PVA	5% MC400	
E9	2,5% PVA	5% MC400	5% PVP
E10	2,5% PVA	5% MC400	5% PVP
E11	2,5% PVA	3% MC1500	
E12	2,5% PVA	3% MC1500	
E13	2,5% PVA	3% MC1500	
E14	2,5% PVA	3% MC1500	5% PVP
E15	2,5% PVA	3% MC1500	5% PVP

* E1-3, E6-8, E11-13: polymers mixed in different proportions

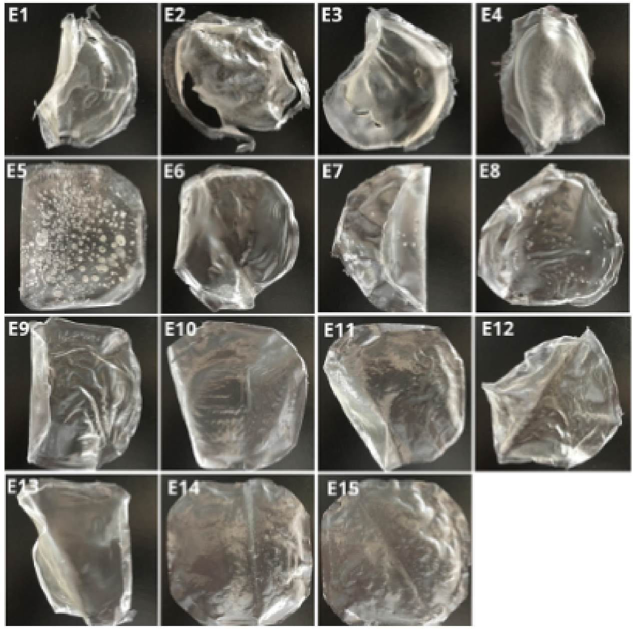

Formulation designation	E1	E2	E3	E4	E5	E6	E7	E8	E9	E10	E11	E12	E13	E14	E15
Polymer agglomerates	+	+	+	+	+	-	-	-	+	-	+	+	-	+	+
Bubbles of air	+	+	+	+	+	-	+	+	-	-	+	+	-	+	+
Flexibility	-	-	-	-	-	-	-	-	-	-	-	-	-	-	-
Clear/transparent	-	-	-	-	-	+	+	+	+	+	+	+	+	-	+
Gloss	+	+	-	-	-	+	+	+	+	+	+	+	+	+	+
Easy to remove from the mold	+	+	+	+	+	+	+	+	+	+	+	+	+	+	+
Disintegration time [h]	5	5	5,5	5,5	5	5,5	5	5,5	5	5,5	5,5	5,5	5,5	5,5	5,5
pH	6,79	6,88	6,85	6,81	6,77	6,80	6,70	6,82	6,98	6,80	6,88	6,81	6,83	6,72	6,72

This diagram clearly shows the process of producing films from a blend of two or three polymer components, taking into account the addition of a plasticiser, and details of the sonication, casting and drying processes of the films, as well as the results of a preliminary assessment of their physico-chemical properties.

1. Purpose:

a. Preparation of films F1 - F9 from mixtures of solutions of two or three polymer components with the addition of 5.0 g of glycerol solution.
b. Evaluation of the physico-chemical properties of the films, including the effect of the addition of plasticizer and moulding compound on the blurring time and structural properties.

2. Fim Process:

a. Prepare polymer solutions according to Table 16 in a laminar chamber.
b. Add 3 ml of water-ethanol solution (water:ethanol 25:75 m/m) to each formulation.
c. Ultrasonication of the mixture in an ultrasonic bath
d. Pouring 40 g or 60 g of the mixture into polystyrene moulds of 92 cm² surface area.
e. Drying in a laboratory dryer for 24 hours at a temperature of 37±1°C.

Formulation designation	Composition of the solution		
F1	2,5% PVA	5% HPMC	
F2	2,5% PVA	5% MC400	
F3	2,5% PVA	5% MC400	
F4	2,5% PVA	5% MC400	
F5	2,5% PVA	5% MC400	
F6	2,5% PVA	5% MC400	5% PVP
F7	2,5% PVA	3% MCA15C	
F8	2,5% PVA	3% MCA15C	5% PVP
F9	2,5% PVA	3% MCA15C	5% PVP

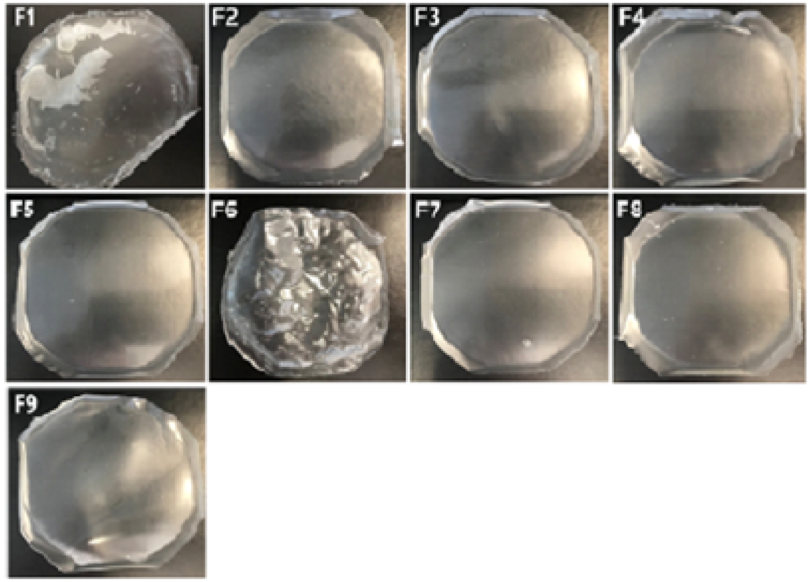

Formulation designation	F1	F2	F3	F4	F5	F6	F7	F8	F9
Polymer agglomerates	+	-	-	-	-	+	-	-	-
Bubbles of air	+	+	+	+	+	+	+	+	-
Flexibility	+	+	+	+	+	+	+	+	+
Clear/transparent	+	+	+	+	+	+	+	+	+
Gloss	+	+	+	+	+	+	+	+	+
Easy to remove from the mold	+	+	+	+	+	+	+	+	+
Mass [g]	60	60	40	60	40	60	40	40	60
Disintegration time [min]	1	22	24	22	24	20	24	22	24
pH	7,09	7,08	7,09	7,07	7,10	7,06	7,04	7,05	7,06

This diagram summarises the key steps and results of an experiment for the optimisation of the composition and production of polymer films.

1. Purpose:

- Mixture of 3 or 4 polymers
- 5.0 g of plasticiser

2. Fim Process:

a. Change in polymer composition.
b. The polymer solutions are pre-mixed and sterilised in the laminar chamber.
c- Pour 30 g of the mixture into polystyrene moulds with a surface area of 92 cm².
d- Freeze and thaw the polymer mixture five times at a temperature of -18 to -22°C.
e- Drying in a laboratory dryer for 48 hours at 32±1°C.

Formulation designation	Composition of the solution			
G1	2,5% PVA	5% HPMC		4% NaCMC
G2	2,5% PVA	5% HPMC	5% PVP	4% NaCMC
G3	2,5% PVA	3% MCA15C		4% NaCMC
G4	2,5% PVA	3% MCA15C	5% PVP	4% NaCMC
G5	2,5% PVA	5% HPMC		4% NaCMC
G6	2,5% PVA	5% HPMC	5% PVP	4% NaCMC
G7	2,5% PVA	5% MC400		4% NaCMC
G8	2,5% PVA	5% MC400	5% PVP	4% NaCMC

G1,5: polymers mixed in different proportions

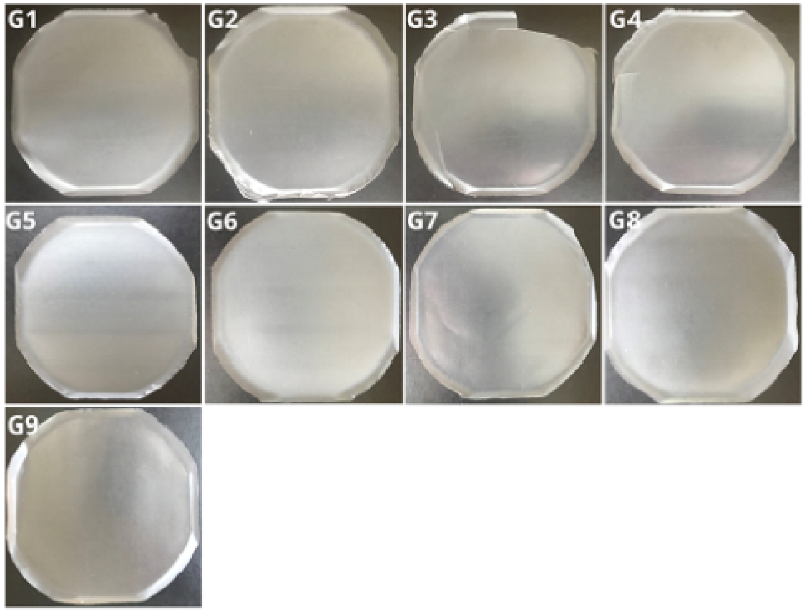

Formulation designation	G1	G2	G3	G4	G5	G6	G7	G8
Polymer agglomerates	-	-	-	-	-	-	-	-
Bubbles of air	-	-	-	-	-	-	-	-
Flexibility	+	+	+	+	+	+	+	+
Clear/transparent	-	-	-	-	-	-	-	-
Gloss	-	-	-	-	-	-	-	-
Easy to remove from the mold	+	+	+	+	+	+	+	+
Mass [g]	30	30	30	30	30	30	30	30
Disintegration time [min]	1	1	5	5	1,5	1,5	5	6
pH	7,45	7,37	7,35	7,30	7,38	7,36	7,32	7,34

The diagram summarises the key steps and results of the experiment to optimise the composition and production of polymer films, including reducing the amount of plasticiser and increasing the amount of NaCMC and the physical cross-linking process

1. Purpose:

- Mixture of 4 polymers
-4.0 g of plasticiser
-Double salt concentration: Na CMC

2. Fim Process:

a. Pour after 45 g.
b. The whole of the technological process is the same as that of the G version.

→

Formulation designation	Composition of the solution			
H1	2,5% PVA	5% MC400	5% PVP	4% NaCMC
H2	2,5% PVA	3% MCA15C	5% PVP	4% NaCMC
H3	2,5% PVA	5% HPMC	5% PVP	4% NaCMC
H4	2,5% PVA	5% HPMC	5% PVP	4% NaCMC

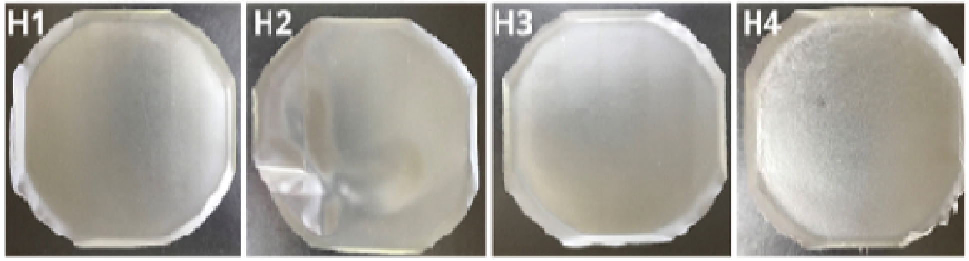

Formulation designation	H1	H2	H3	H4
Polymer agglomerates	-	-	-	+
Bubbles of air	-	-	-	-
Flexibility	+	+	+	+
Clear/transparent	-	-	-	-
Gloss	+	+	+	+
Easy to remove from the mold	+	+	+	+
Mass [g]	45	45	45	45
Disintegration time [min]	20	20	18	15
pH	7,43	7,40	7,41	7,34

The diagram summarises the key steps and results of the experiment to optimise the composition and production of polymer films, including reducing the amount of plasticiser and increasing the amount of NaCMC and the physical cross-linking process

1. Purpose:

- Mixture of 4 polymers
-3.0 g of plasticiser
-Double salt concentration: Na CMC

2. Fim Process:

a. The whole of the technological process is the same as that of the H version.

→

Formulation designation	Composition of the solution			
I1	2,5% PVA	5% MC400	5% PVP	4% NaCMC
I2	2,5% PVA	3% MCA15C	5% PVP	4% NaCMC
I3	2,5% PVA	5% HPMC	5% PVP	4% NaCMC

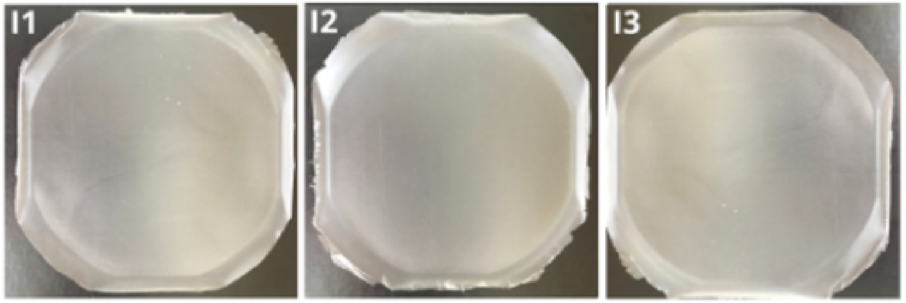

Formulation designation	I1	I2	I3
Polymer agglomerates	-	-	-
Bubbles of air	-	-	-
Flexibility	+	+	+
Clear/transparent	-	-	-
Gloss	+	+	+
Easy to remove from the mold	+	+	+
Mass [g]	45	45	45
Disintegration time [min]	24	24	20
pH	7,17	7,19	7,28
